# A Rare Case of Spontaneous Anterior Abdominal Wall Necrotizing Soft Tissue Infection Caused by Finegoldia magna

**DOI:** 10.7759/cureus.19685

**Published:** 2021-11-18

**Authors:** Nimisha Thomas, Dipali Taggarsi, Prudhvi Dasari, Roger Rathna

**Affiliations:** 1 Critical Care Medicine, St. John's Medical College Hospital, Bangalore, IND

**Keywords:** necrotizing fasciitis, nsti, pelvic abscess, gram positive anaerobic cocci, necrotizing soft tissue infection, abdominal wall necrotizing fasciitis, acute surgical abdomen, finegoldia magna

## Abstract

Necrotizing fasciitis is a highly invasive disease characterized by rapidly worsening inflammation of the fascia associated with necrosis of the subcutaneous tissue. It is a rare, life-threatening disease and needs early diagnosis through acute clinical awareness. It warrants urgent and aggressive surgical management. We report a rare and fatal case of spontaneous necrotizing soft tissue infection (NSTI) of the anterior abdominal wall caused by *Finegoldia magna* in a chronic diabetic patient. The initial presentation and radiological features suggested a pelvic abscess. Other acute abdomen differentials were also considered, and the patient underwent an exploratory laparotomy where a necrotic anterior abdominal wall with copious purulent secretions was noted. The organism *Finegoldia magna* was identified from the necrotic tissue sample with other cultures coming back negative. The risk posed by NSTIs is very high, and hence though the incidence is low, clinicians should be aware of the potential dangers of this disease to avoid delays in initiating appropriate treatment.

## Introduction

Necrotizing fasciitis is a relatively rare, potentially fatal soft tissue infection that needs early diagnosis followed by urgent and aggressive surgical management. The first account of this was by Hippocrates circa 500 BC [1], and subsequently, it was described as “hospital gangrene” by British Naval surgeons in the 18th and 19th centuries [2]. Various other terms had been used to describe the condition till 1951 when Dr. Wilson introduced the term necrotizing fasciitis. Fascial necrosis, irrespective of the causative organism, was pathognomonic of the condition and considered a defining step in the pathophysiology of the disease process [3]. More recently, the term necrotizing soft tissue infection (NSTI) has been advocated, since all soft tissue necrotizing infection implies similar pathophysiology, approach, and management irrespective of its categorization [4].

This condition requires a high index of suspicion for diagnosing and treating in a timely manner. It may be classified based on anatomy, depth, and causative microbial organisms. It is most commonly polymicrobial in etiology with gram-positive cocci being the most implicated. *Finegoldia magna*, previously known as *Peptostreptococcus magnus*, is a gram-positive bacterium inhabiting the skin, gastrointestinal, and genitourinary tracts and is often considered a contaminant. However, it has been speculated to be a neglected cause of toxic shock syndrome [5], and there seem to be extremely few reports in the medical literature describing NSTI of the abdominal wall caused by *F. magna* [6].

## Case presentation

History and initial presentation

A 50-year-old tubectomised lady, who was a known case of diabetes mellitus for four years (hemoglobin A1C [HbA1c]: 7.3), came with complaints of abdominal pain, distension, vomiting, and fever of three days duration, along with decreased urine output for the past one day. The pain was dull aching and more on the left side. The patient also complained of two to three episodes of vomiting per day, which was non-bilious and not bloodstained but did not report obstipation or constipation. She consulted a local hospital where an ultrasonography (USG) was done, which showed a focal peritoneal collection in the suprapubic region. She was therefore referred to a tertiary center and presented at our emergency room (ER).

On arrival at our ER, the patient was noted to be stable but appeared unwell. Her heart rate was 115 beats per minute (bpm), and her blood pressure recorded was 110/70 mm Hg. On per abdominal examination, there was distension noted with guarding and tenderness in the left iliac and hypochondriac areas. There were no excoriations, or open wounds suggestive of an entry point of infection, and no scar other than her tubectomy scar. Per speculum examination findings were unremarkable. A per vaginal examination revealed a vague mass, minimally tender corresponding to 14 weeks. Cervical motion tenderness or forniceal tenderness was not elicited.

Investigations

**Figure 1:**
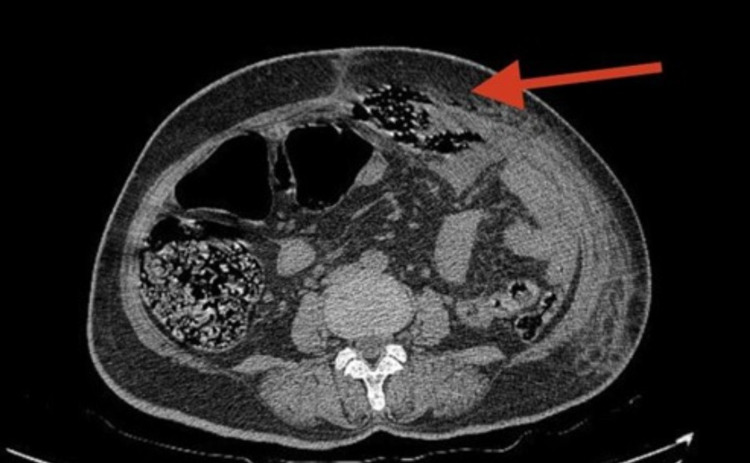
Large collection (red arrow) is noted in pelvis extending into right adnexa and anterior abdominal wall

At presentation, blood gas analysis was generally normal except for lactate of 4.6 mmol/L. Renal function and complete blood counts were within the normal range. A contrast-enhanced computed tomography (CECT) of the abdomen was done to localize and quantify the collection and rule out serious causes like perforated viscous, ischemic bowel, and volvulus. CECT abdomen revealed a large collection in the pelvis (Figure 1) with enhancing areas extending into the right adnexa and anterior abdominal wall with pneumoperitoneum, with no obvious communication with bowel loop. Clinically, the initial differentials considered were pyosalpinx, adnexal cyst, ovarian malignancy, and ectopic pregnancy. Radiologically, adnexal, omental, or neoplastic causes were suggested. Pregnancy was ruled out with a urine pregnancy test, and tumor markers sent for malignancy were negative.

Treatment

**Figure 2:**
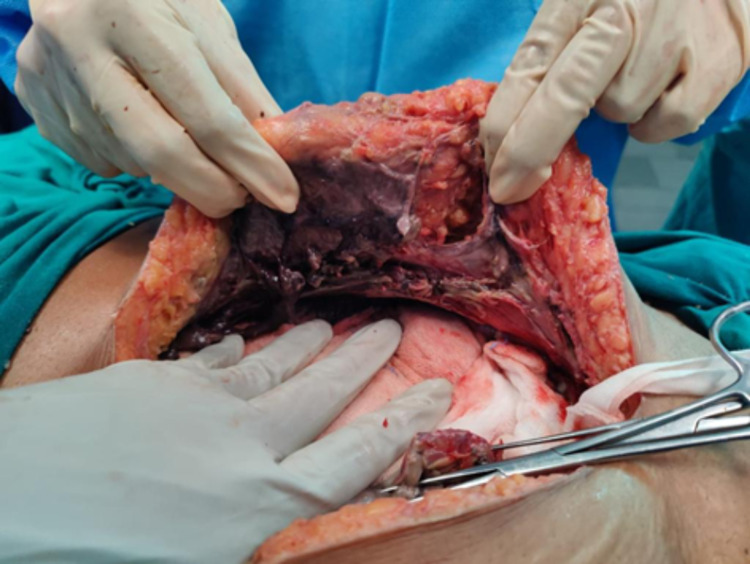
Blackish discoloration at the umbilical area, indicating necrosis of the muscles of the anterior abdominal wall

A preliminary diagnosis of pelvic abscess was made, and she was started empirically on metronidazole and piperacillin-tazobactam. She was taken up for an emergency laparotomy by the obstetrics and gynecology team after 10 hours of initial presentation. A midline vertical abdominal incision was made. Dissection revealed blackish discoloration in the umbilical area, and the general surgery team was called to debride the same. The incision was deepened to the rectus sheath, and a necrotic rectus sheath was noted (Figure 2). The uterus, bilateral ovaries, and tubes appeared to be normal. A purulent collection of approximately 1 L, along with sloughed out rectus sheath, rectus abdominis muscle, and transverse abdominis muscle, was noted, while bowel appeared normal. The collection was drained, and temporary closure was done with an intraperitoneal and subcutaneous drain in situ. Necrotic tissue that was excised was immediately sent for histopathological evaluation as well as culture and sensitivity. Intraoperative blood gas analysis was suggestive of severe metabolic acidosis with lactates of 4.5 mmol/L; hence, elective extubation was deferred. The patient was shifted to the intensive care unit (ICU) for mechanical ventilation, a possible requirement of hemodialysis, and further management.

Postoperative care

The patient was continued on empirical antibiotics piperacillin-tazobactam and metronidazole. Meanwhile, the anaerobic culture sent of the tissue sample collected intraoperatively grew a single organism, *Finegoldia magna*. All other cultures of bronchoalveolar lavage (BAL), blood, urine, and tissue, including mycobacterial and fungal cultures, were negative. Histopathological examination of the tissue sent intraoperatively from the anterior abdominal wall showed fibromuscular and fibroadipose tissue chiefly showing necrosis.

Despite initial therapy, the patient had persistently high lactates because of which clindamycin was added on the third postoperative day. The patient was taken up for a relook laparotomy 60 hours after the first surgery. Intraoperatively, 350 ml purulent collection was drained, slough in transverse abdominis muscle was debrided, and slough extending up to the fourth intercostal space was noted. Pelvic and left intercostal drains were placed, and the patient was shifted back to the ICU. Despite extensive source control and antibiotic therapy, the patient continued to deteriorate and developed septic shock. She succumbed to her illness on the seventh day of ICU admission.

## Discussion

*Finegoldia magna* was previously referred to as *Peptostreptococcus magnus* and is a member of the Clostridiales family. It is a pleomorphic, gram-positive, non-spore-forming obligate anaerobe that is an important opportunistic pathogen among the many gram-positive anaerobic commensals that colonize the skin and mucosal surfaces. Gram-positive anaerobic cocci (GPAC), though frequently isolated from cultures, being a major component of the human flora, are insufficiently studied, and their effect as a pathogen is very little recognized. They have a repeatedly modified classification and confusing taxonomy with obvious unavailability of clear guidelines for characterization which has been discouraging for studies on the role of these organisms in health and disease [7].

*F. magna* bacteria are strict anaerobes during multiplication and aerotolerant in clinical isolates and best grown from aspirates or tissue that reach the laboratory promptly. The bacteria has two main virulence factors, FAF and SuFA, that exhibit primarily adhesive and proteolytic properties, respectively. These allow the breakdown of the basement membrane and infect deeper layers of the skin and tissues, facilitated by an epithelial breach or immunocompromised state [8]. Rosenthal et al. [5] hypothesized in 2012 that *F. magna* is a neglected causality of toxic shock syndrome and that the general importance of this organism may be underestimated. It has a capability to initiate a cascade of downstream immunoregulatory phenomena, which most likely are mediated by protein L activity in conjunction with other virulence factors like albumin-binding protein, thereby letting this commensal act as an opportunistic pathogen [5]. *F. magna* acts by the activation of human neutrophils, through its soluble proteins FAF and protein L, thus inducing a pro-inflammatory response, and these proteins also block the effect of antibacterial peptides or proteins. Collectively these mechanisms equip it with a higher pathogenic potential compared to other GPAC [9].

*F. magna* has been implicated in many serious infections, like joint and prosthetic infections, gynecological infections, abdominal infections, diabetic foot ulcers, necrotizing fasciitis, and all abscesses. To the best of our knowledge, the only other case describing abdominal wall necrotizing fasciitis caused by this organism was described by Begaj et al. [6], where the patient initially noticed a lump that subsequently progressed to cellulitis which did not resolve with antibiotics, and the diagnosis of necrotizing fasciitis was made on her 11th day of symptoms. The patient was taken up for debridement of the necrotic area three times and was discharged seven days after initial hospital admission. She was followed up after six weeks, and the postoperative wound was noted to be smaller with adequate granulation [6].

In our case, the patient had a more rapid course progressing over three days to form a large pelvic collection with abdominal extension. While the immunocompromised state of the patient due to diabetes probably contributed to the causation of this opportunistic infection, there is no history suggestive of any skin infections, intra-abdominal causes like the ruptured appendix, or trauma preceding the event. The possibility of NSTI developing from an internal source of infection may be considered. It could also be speculated that symptoms of abdominal wall cellulitis might have been missed or unnoticed, and the course may have been longer than the three-day duration assumed at presentation. The clinical presentation was more suggestive of an abscess of adnexal or omental origin, and a malignant cause was also considered as one of the differentials. The patient presented in a very late stage of the disease process leading to the unfortunate outcome despite aggressive management. 

This goes on to emphasize that early diagnosis, a high level of clinical suspicion, and early aggressive management are the only effective tools against NSTI. The mortality reduces dramatically from 38% for delayed incomplete debridement to 4% for early complete debridement according to Bilton et al. [10]. The treatment recommended in an anterior abdominal NSTI is antibiotic therapy followed by serial debridement, ICU care with nutritional support (equivalent to burns and major trauma), and close wound monitoring. Vacuum-assisted closure therapy (VAC) is being increasingly considered superior for fast and effective wound closure [11].

## Conclusions

Necrotizing soft tissue infections (NSTIs) are potentially fatal owing to the rapid progression of the disease and clinical deterioration. It is of paramount importance to arrive at the diagnosis in time, as expeditious treatment favors better outcomes. There may not necessarily be a skin breach for their development but can occur as an extension of a focus of infection from within the body. The diagnostic challenge posed by NSTIs should be recognized, and a high index of suspicion should be maintained for the same. The cost of delays in arriving at the diagnosis is huge and can prove fatal.
